# Novel Gliclazide Electrosprayed Nano-Solid Dispersions: Physicochemical Characterization and Dissolution Evaluation

**DOI:** 10.15171/apb.2019.026

**Published:** 2019-06-01

**Authors:** Khosro Adibkia, Solmaz Ghajar, Karim Osouli-Bostanabad, Niloufar Balaei, Shahram Emami, Mohammad Barzegar-Jalali

**Affiliations:** ^1^Research Center for Pharmaceutical Nanotechnology and Faculty of Pharmacy, Tabriz University of Medical Sciences, Tabriz, Iran.; ^2^Drug Applied Research Center and Faculty of Pharmacy, Tabriz University of Medical Sciences, Tabriz, Iran.; ^3^Students Research Committee, Tabriz University of Medical Sciences, Tabriz, Iran.; ^4^Research Center for Pharmaceutical Nanotechnology, Tabriz University of Medical Sciences, Tabriz, Iran.; ^5^Faculty of Pharmacy, Tabriz University of Medical Sciences, Tabriz, Iran.; ^6^Pharmaceutical Analysis Research Center, Tabriz University of Medical Sciences, Tabriz, Iran.

**Keywords:** Amorphous solid dispersions, Eudragit® RS100, Electrospray, Gliclazide, In vitro evaluation, Nanocrystalline, Polyethylene glycol 6000

## Abstract

***Purpose:*** In the current study, electrospraying was directed as a novel alternative approach to improve the physicochemical attributes of gliclazide (GLC), as a poorly water-soluble drug, by creating nanocrystalline/amorphous solid dispersions (ESSs).

***Methods:*** ESSs were formulated using Eudragit® RS100 and polyethylene glycol (PEG) 6000 as polymeric carriers at various drug: polymer ratios (i.e. 1:5 and 1:10) with different total solution concentrations of 10, 15, and 20% w/v. Morphological, physicochemical, and in-vitro release characteristics of the developed formulations were assessed. Furthermore, GLC dissolution behaviors from ESSs were fitted to various models in order to realize the drug release mechanism.

***Results:*** Field emission scanning electron microscopy analyses revealed that the size and morphology of the ESSs were affected by the drug: polymer ratios and solution concentrations. The polymer ratio augmentation led to increase in the particle size while the solution concentration enhancement yielded in a fiber establishment. Differential scanning calorimetry and powder X-ray diffraction investigations demonstrated that the ESSs were present in an amorphous state. Furthermore, the in vitro drug release studies depicted that the samples prepared employing PEG 6000 as carrier enhanced the dissolution rate and the model that appropriately fitted the release behavior of ESSs was Weibull model, where demonstrating a Fickian diffusion as the leading release mechanism. Fourier-transform infrared spectroscopy results showed a probability of complexation or hydrogen bonding, development between GLC and the polymers in the solid state.

***Conclusion:*** Hence the electrospraying system avails the both nanosizing and amorphization advantages, therefore, it can be efficiently applied to formulating of ESSs of BCS Class II drugs.

## Introduction

One of the prevalent chronic metabolic disorders affecting seriously peoples’ health, is type II (non-insulin dependent) diabetes mellitus (T2DM), identified by pancreatic β-cell inadequate secretion of insulin and/or abnormal secretion of glucagon and/or tissue responses reduction to insulin.^1^ It has been reported that almost about 90% of diabetic cases suffer from T2DM where an uncontrolled DM could cause numerous long-term complications such as nephropathy, retinopathy, cardiovascular disorders (CVDs), and peripheral neuropathy.^2^ Recently, along with lifestyle changing which play a vital role in controlling T2DM, metformin (MTF) has been the primary oral therapy for T2DM. However, some side effects including vomiting, nausea, abdominal pain (that happen in around 20% of cases), diarrhea, and serve/ rarely lactic acidosis are associated with the use of this drug.^3^ Patients with contraindication to MTF can take sulphonylureas as an alternative.^4^ Gliclazide (GLC) (C_15_H_21_N_3_O_3_S) is a 2^nd^ generation of hypoglycemic sulphonylurea that can be used to treat T2DM.^5^ In comparison with other sulphonylureas, GLC has less side effects, lower risks of CVDs and hypoglycaemia with comparable efficacy, due to its shorter half-life, exclusive antioxidant characteristics, selective inhibitory action regard pancreatic K_ATP_ channels by insulin secretion stimulation from β cells of pancreas, and some other useful haemobiological properties.^6,7^ The mechanism of GLC in protecting the vasculature is based on its role as a free radical scavenger, increasing fibrinolysis and tissue plasminogen activator which leads to platelet and plasma lipids function improvements.^8,9^ These advantages candidate GLC as a good long-term medication for T2DM treatment, as well as place it at essential medicines list of the world health organization. However, GLC is a weak acid (pKa 5.8) which has low water solubility (55 µg/mL). This drug indicates slow absorption rate at gastrointestinal tract (GIT) with a variable bioavailability.^10,11^ The drug low dissolution rate within the prepared formulation and/or its limited permeability through GIT membrane are common reasons of the drug poor absorption rate. This behavior of GLC, at least in part, can be referred to its powder hydrophobicity as demonstrated by low surface wetting of its powders in contact with water. For class II drugs based on Biopharmaceutical Classification System (BCS) (with limited water solubility and high permeability), the dissolution rate in the GIT usually controls the oral absorption rate.^12^ Therefore, along with permeability, a drug dissolution rate and/or solubility are key factors that determine its oral bioavailability.^13^ This evidence proposes that the enhanced GIT absorption of GLC could be acquired using its augmented release formulations which increase the dissolution rate and consequently improve bioavailability. Various methods have been applied to formulate GLC with enhanced dissolution rate such as micronization,^14^ multicomponent crystals fabrication,^15^ neutralization and recrystallization,^16^ and solid dispersion (SD).^17^

SD, as one of the mostly applied methods to dissolution enhancement of class II drugs, deals with a group of solid components including of at least two distinct elements (a hydrophobic drug and a hydrophilic/amphiphilic carrier).^18-20^ Conventional basic procedures used efficiently to produce SDs of different drugs are melt crystallization, lyophilization,^21^ spray drying,^22^ solvent evaporation, hot melt extrusion, and cogrinding.^19,20,23^ Electrospraying or electrospinning is an emerging alternative method for SDs preparation.^24^ The efficacy of the electrospraying technique for dissolution enhancement of different drugs with low water solubility including atorvastatin calcium, ezetimibe,^24^ propranolol hydrochloride (HCl),^25^ raloxifene HCl,^26^ anticancer drugs,^27^ and azithromycin^28^ have been demonstrated by many authors.

Electrospraying (ECS)/electrospinning is a flexible approach with the potency of producing various formulations in the range of micro-nano size for a broad appeals scope in the pharmacy sector.^29,30^ This economic, easily adjustable, and one-step system basically uses an electric power to atomize a polymer-drug solution. In another word by imposing a high voltage electrical force, the polymer-drug solution droplets from a syringe will jet-out (atomize) and form micro/nano sized particles/fibers on a grounded screen placed under the tip of the syringe.^25,31^ The applied voltage, flow rate of the polymer-drug solution, and deposition distance as the system variables, surface tension, electrical conductivity, viscosity, and density as the polymer physical features, polymer to drug ratios and jetting behavior of the system (polymer-drug solution) are the affecting and controlling parameters that lead to nanofibers or nanoparticles (nanobeads) formation.^32,33^

The dissolution rate augmentation mechanisms may be related to size reduction or aggregation absence of drug crystallites, the polymer solubilization influence, dispersibility and wettability enhancement of the drug, phase transformation of the drug from crystalline to amorphous state, and the dissolution of the drug molecules in the hydrophilic polymer matrix.^19,34,35^ Eudragit^®^ RS100 (Eudr) is a hydrophilic water-insoluble copolymer of poly (ethylacrylate, methyl-methacrylate and chlorotrimethyl-ammonioethyl methacrylate) containing quaternary ammonium groups (4.5- 6.8 %) with particular attributes such as non-toxicity, high permeability, good stability and aqueous media swelling ability.^25,36^ Polyethylene glycol (PEG) as the mostly used polymer for SDs preparation has special advantages including good solubility in water and numerous organic solvents, quite low melting point, solubilizing ability of some combinations,^37^ and wettability augmentation.^13^ Considering aforementioned parameters it seems that preparing nano-solid dispersions (NSDs) of GLC with Eudr and PEG may be beneficial to solve solubility, stability, dissolution, and bioavailability issues. Therefore, in the present study Eudr and PEG 6000 were used as two appropriate candidates to prepare GLC NSDs conducting ECS method, with different drug to polymer ratios at various solution concentrations to enhance physicochemical characteristics of GLC.

## Materials and Methods

### Materials

GLC, PEG 6000, potassium phosphate monobasic and sodium hydroxide were obtained from Merck (Germany). Eudragit^®^ RS100 and acetone were purchased from Degussa (Darmstadat, Germany) and Duksan (South Korea), respectively. All other chemical materials were analytical grade.

### Electrospraying procedures

GLC- Eudr and GLC- PEG formulations were developed applying a customized ECS apparatus (Fanavaran Nano-Meghyas, Tehran, Iran). Briefly, GLC-Eudr solutions with drug: polymer ratios of 1:5 and 1:10 at total solution concentrations of 10, 15, and 20 % (w/v) were processed by co-dissolving of GLC and Eudr in acetone at ambient temperature. Furthermore, another sample was prepared with GLC: PEG 6000 ratio of 1:5 at a total solution concentration of 10 % (*w/v*) as the same procedure of GLC- Eudr samples (Table 1).

**Table 1:** Key formulation composition of the electrosprayed gliclazide samples

**Formulation**	**Components**	**Drug to polymer ratios**	**Total solution concentration % (w/v)**
F1	Gliclazide- Eudragit^®^RS100	1:5	10
F2	Gliclazide- Eudragit^®^RS100	1:5	15
F3	Gliclazide- Eudragit^®^RS100	1:5	20
F4	Gliclazide- Eudragit^®^RS100	1:10	10
F5	Gliclazide- Eudragit^®^RS100	1:10	15
F6	Gliclazide- Eudragit^®^RS100	1:10	20
F7	Gliclazide- Polyethylene Glycol 6000	1:5	10

Formulation = F.

The prepared solutions were jetted, applying a 25 kV voltage to the syringe needle (gauge 29) attached to a polyethylene made ring shaped capillary tube (inner diameter of 0.1 mm). By applying the voltage, the solutions were flowed towards a grounded collector screen coated with polytetrafluoroethylene and formed GLC- Eudr/ GLC- PEG 6000 SDs. The distance between the syringe tip and grounded screen, and the injection rate of the feed solution were kept at 10 cm and 5 mL/h, respectively.

### Field emission scanning electron microscopy (FE-SEM)

The processed samples morphology was evaluated using a field emission scanning electron microscope (MIRA3, Tescan Co., Brno, Czech) at operational 20 kV condition. The electrosprayed samples (ESSs) were coated with a thin gold layer (about 150 Å in thickness) using gold sputtering apparatus (Emitech K550, Kent, UK) prior evaluation by FE-SEM. The average diameters of ESSs were assessed directly from FE-SEM figures by measuring the samples diameters at above 50 points conducting Digimizer image analysis software. The determined diameters were represented as ‘‘mean Feret diameter ± standard deviation’’. Measuring of a particle size along a particular direction is called the Feret/Feret’s diameter. Generally, it can be described as the space between two parallel tangential lines that perpendicularly limiting the particle to that direction. This method is applied to measure particle sizes in microscopy, where a 3-dimensional particle is projected on a 2-dimensional plane.^38^

### Differential Scanning Calorimetry (DSC)

A DSC 60 (Shimadzu, Kyoto, Japan) was benefited to analyze thermal behaviors of pure GLC, Eudr, PEG 6000, physical mixtures (PMs), and ESSs. In this regard, 5 mg of each samples was placed in a sealed aluminum pan then the samples thermal behavior was assessed in the range of 25–220°C at a scan rate of 20°C/min and analyzed by TA60 software. As standard and reference samples, indium and aluminum oxide powders were benefited, respectively.

### Powder X-ray diffraction (PXRD)

The pure GLC, Eudr, PEG 6000, PMs and ESSs PXRD patterns were measured conducting an X-ray diffractometer D 5000 (Siemens, Munich, Germany) at step size, 2θ angle range, and scanning rate of 0.02°,5–30°and 0.6°/min, respectively. The operational parameters were Cu K_α_ radiation (λ=1.5405 Å) at 40 kV, 30 mA.

### Fourier transform infrared spectroscopy (FTIR)

The probable chemical interactions between the drug and studied polymers were investigated using the FTIR spectrophotometer (Shimadzu 43000, Kyoto, Japan). For performing the analysis, the pure GLC, Eudr, PEG 6000, PMs and ESSs were compacted in a disc shape by KBr disk method and studied at a resolution of 2 cm^-1^ with average spectra of 32 scans in scanning range of 4000–600 cm^-1^.

### In vitro drug release

The dissolution studies of pure GLC, PMs, and ESSs were carried out by means of USP paddle method (apparatus II). In this regard, samples corresponding to 20 mg of GLC were positioned in the vessel containing 300 mL of phosphate buffer (pH 6.8) under rotational motion (50 rpm) at 37±0.2°C. At prearranged spans, 3 mL of the treated solutions was removed and substituted with an equal volume of fresh buffer for the purpose of maintaining a constant volume. The removed solution was filtered using a membrane made of cellulose acetate (pore size 20 nm, Whatman, Kent, UK) and analyzed by a UV spectrophotometer (Shimadzu, Kyoto, Japan) at a wavelength of 228 nm to assess the drug cumulative release graphs. The average values of three assessments were used.

### Drug release assessment

The DD-solver computer software was benefited for quantitatively evaluation of the drug release kinetics and its dissolution data.^39^ The influence of ECS process besides the effects of polymer ratios on the dissolution behaviors of GLC were determined by computing t_50%_ (demanded time for releasing 50% of the drug), Q_30min_ and Q_120min_ (the dissolved drug percent within 30 and 120 min, respectively) magnitudes. Various models including zero-order, first-order, Higuchi, Korsmeyer-Peppas, Hixson-Crowell, and Weibull were applied to fit the release data. In order to determine how each model could properly fit the data, the adjusted coefficient of determination (R^2^_adj_) as statistical criteria and the model selection criterion (MSC) were calculated. The highest R^2^_adj_ and the largest MSC of a model nominated it as the best fitting model.

## Results and Discussion

### Morphological evaluation of ESSs

**Figure 1:**
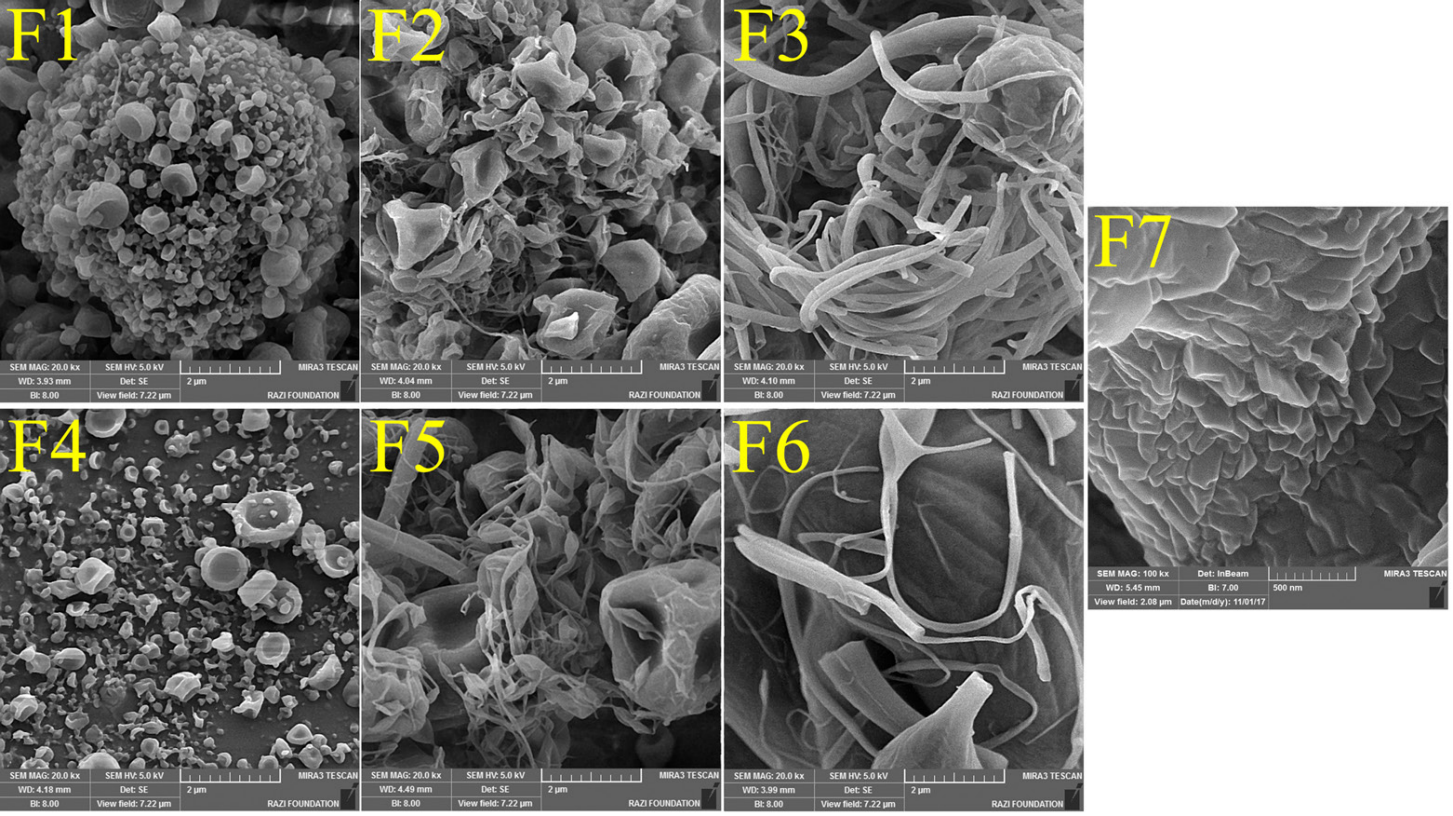
Field emission scanning electron microscopy images of the gliclazide – Eudragit^®^ RS100 (F1-F6) and Polyethylene glycol 6000 (F7) electrosprayed nano-solid dispersions with (F1) drug: polymer ratio of 1:5 at the total solution concentration of 10% w/v, (F2) 1:5-15% w/v, (F3) 1:5-20% w/v, (F4) 1:10-10% w/v, (F5) 1:10-15% w/v, (F6) 1:10-20% w/v and (F7) 1:5-10% w/v.

The morphology and size distribution of GLC-Eudr and GLC-PEG 6000 ESSs are shown in Figure 1. As literature review revealed the ESSs morphological features as well as their size distribution are crucial characteristics impress drug delivery mechanisms and its effectiveness, where these parameters depend on the ambient, solution, and operation variables.^24,25,40^ The operation variables, including the feeding rate, applied voltage, and the distance between syringe/ nozzle tip and grounded screen along with ambient factors were fixed in regard to our preliminary analyzes. In the current study, the solution characteristics such as polymer and solution concentrations were selected as variable parameters.

The ECS solution concentration along with polymer ratio are the chief elements in controlling the particles morphology and size.^24^ Corresponding to FE-SEM images, it was recognized that the lower solution concentrations (i.e. 10 and 15% w/v) led to nanobeads/nanoparticles in a concave shape (Figure 1 (F1, F2, F4, F5)), whereas nanofibers formation resulted in the higher solution concentrations (i.e. 20% w/v) (Figure 1 (F3, F6)). Table 2 indicates relevant average particles and fibers diameter of the prepared formulations.

**Table 2:** Average diameters of electrosprayed beads/particles and fibers

**Formulation**	**Bead/Particle and Fiber Diameter (nm)**
F1 (1:5; 10%); Gliclazide-Eudragit® RS100	156.05 ± 32
F2 (1:5; 15%); Gliclazide-Eudragit® RS100	319.90 ± 39
F3 (1:5; 20%); Gliclazide-Eudragit® RS100	411.00 ± 46
F4 (1:10; 10%); Gliclazide-Eudragit® RS100	213.30 ± 39
F5 (1:10; 15%); Gliclazide-Eudragit® RS100	398.50 ± 60
F6 (1:10; 20%); Gliclazide-Eudragit® RS100	512.00 ± 69
F7 (1:5; 10%); Gliclazide-PEG 6000	-

The observed particulate properties could be attributed to the viscoelastic forces enhancement with increasing the concentration of the ECS solution, which could prevail the surface tension and induce formation of the fibers, while nanobeads development occurs at high surface tension of the solutions that disperse the liquid to separate droplets. Comparable results have been reported in other studies.^24,25,31,41^ Additionally, geometric characteristics of the ESSs could be influenced by the electrical conductivity and solution viscosity alterations. In other words, reducing the solution viscosity as well as raising the charge density develops smaller beads or fibers.^42^ In this regard, the larger nanobeads development by augmenting the drug: polymer ratio could be linked to the electrical conductivity reduction of the processed solution at the high polymer ratios. It is worth to note that, the ESSs prepared using PEG 6000 as a carrier with drug: polymer ratio of 1:5 at a total solution concentration of 10% (*w/v*) (Figure 1 (F7)) resulted a merged nanobeads that this phenomenon could originate as a consequence of the operative distance between grounded collector screen and the syringe tip being too low. Thereby, there is an inadequate area for the viscous solution to stretch at the syringe/nozzle, inducing insufficient solvent evaporation to create spread particles.^31^

### Differential scanning calorimetry

**Figure 2:**
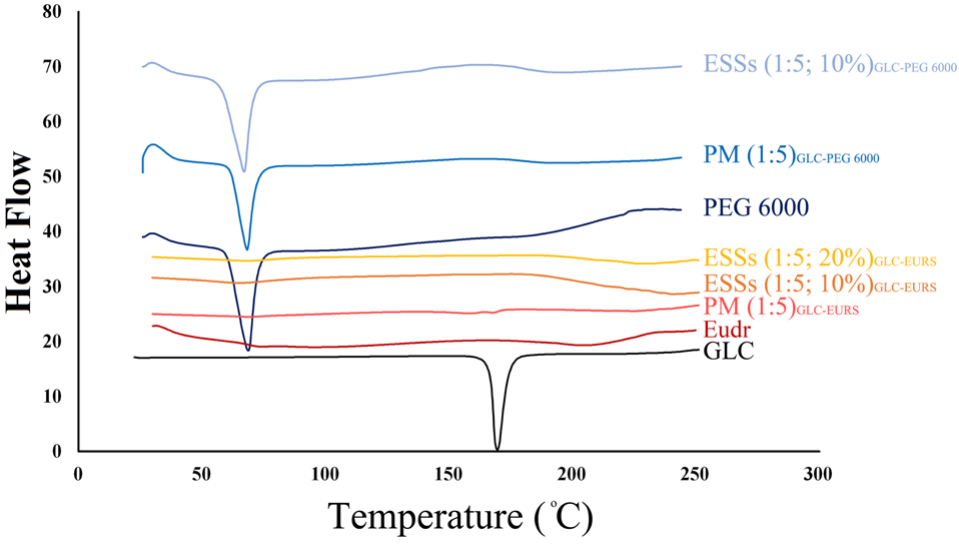
Differential scanning calorimetry thermograms of the pure gliclazide (GLC), Eudragit® RS100 (Eudr), Polyethylene glycol 6000 (PEG 6000), physical mixture (PM) and electrosprayed nano-solid dispersions (ESSs) with the drug: polymer ratio of 1:5 at total solution concentrations of 10% and 20% (w/v).

The thermograms of pure GLC, Eudr, PEG 6000, PMs, and ESSs were surveyed by DSC (Figure 2). Analyzing the thermal behavior of GLC revealed an endothermic peak around 170°C related to its melting point with the corresponding enthalpy of fusion (ΔH) of 171.8 J/g.^13,18^ Scanning of PEG 6000 revealed an endothermic peak at 61.9°C with a ΔH of 188.6 J/g,^35^ where an amorphous attitude was depicted in the thermogram of Eudr with a glass transition temperature of 58.44°C.^25^ Thermograms of ESSs prepared by using Eudr indicated the absence of GLC melting peak suggesting that GLC was entirely solubilized in the applied polymers, or its crystalline structure transformed to an amorphous state.

Furthermore, the endothermic peak of the drug was absent in the PMs that implying the solubilizing and/or dilution influence of the polymers on GLC and/or the drug-polymers, heat prompted interactions.^24^ On the other hand, compared to pure PEG 6000, the ESSs prepared by PEG revealed PEG 6000 melting peak at quite lower temperature. These results are in good agreement with previous reports.^13,24,25^

### Powder X-ray diffraction (PXRD) evaluation

**Figure 3:**
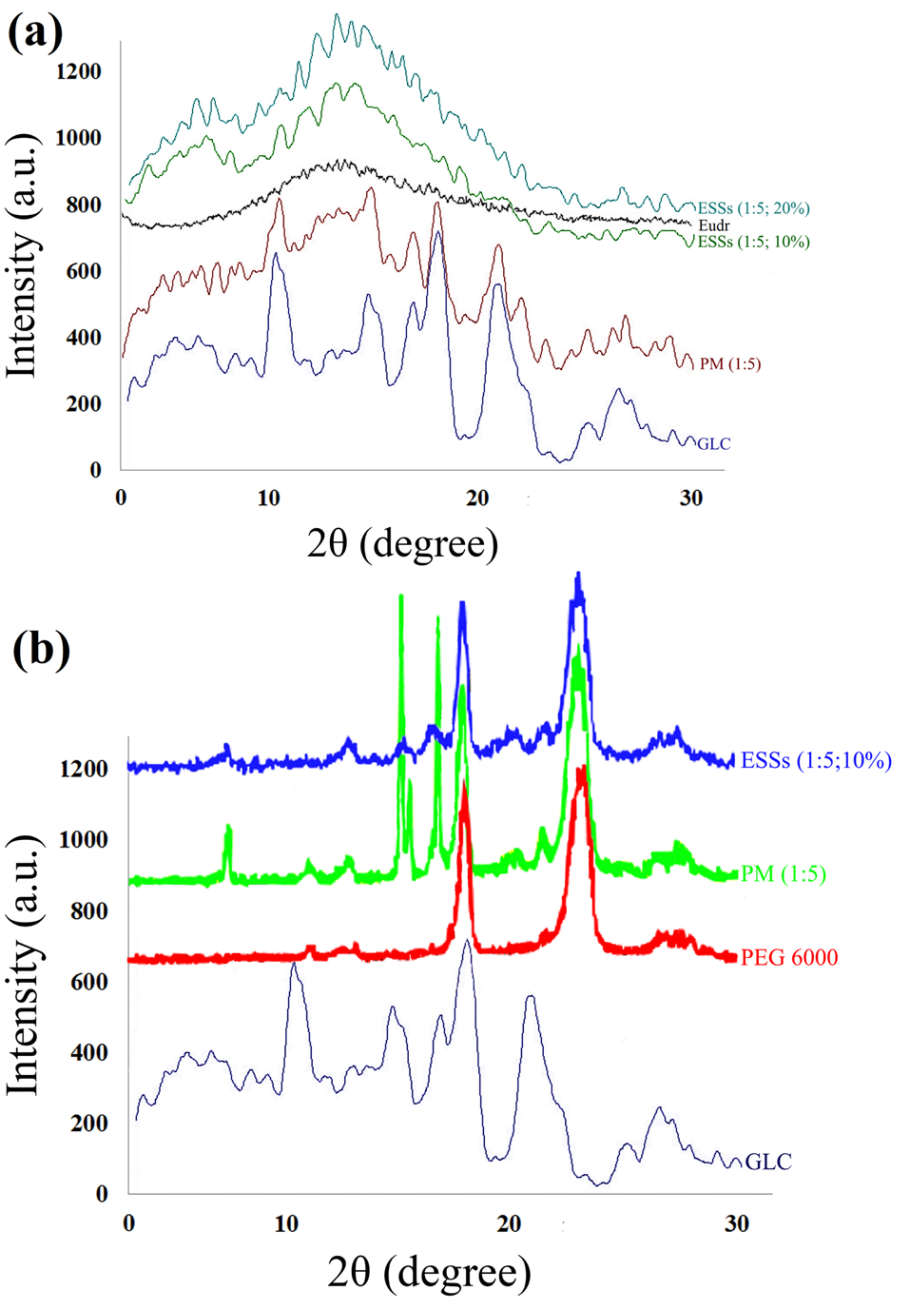
Powder X-Ray Diffraction patterns of (a) pure gliclazide (GLC), Eudragit® RS100 (Eudr), physical mixture (PM) and electrosprayed nano-solid dispersions (ESSs) with the drug: polymer ratio of 1:5 at total solution concentrations of 10% and 20% (w/v) and (b) GLC, Polyethylene glycol 6000 (PEG 6000), PM and ESSs 1:5-10% w/v.

Figure 3 indicates the diffraction patterns of the pure drug, Eudr, PEG 6000, PMs, and ESSs. GLC diffraction pattern represented its crystalline structure as confirmed by several sharp, distinguished diffraction peaks detected at 2θ angles of 10.59°, 14.98°, 17.21°, 17.85°, 18.15°, 22.07°, 25.42°, 26.25°, 26.75°, and 29.51° that is in good agreement with previous studies.^10,13,18^ Two distinctive peaks of PEG 6000 with highest intensity were identified at 2θ angles of 19.41° and 23.34° (Figure 3b), where the lack of any representative peaks in the PXRD spectrum of Eudr uncovered its amorphous nature (Figure 3a).

The dissolution rate of a drug could be affected by its degree of crystallinity, where the drug in metastable or amorphous state will show the highest dissolution rate due to its considerable molecular activity and superior internal energy that raise the thermodynamic attributes in comparison with that of crystalline substances.^43^ Considering Figure 3, it is visible that PMs along with ESSs showed certain shifts and variations in the position of diffraction peaks of GLC at PXRD spectrums. The distinguished peaks of pure GLC at 2θ angles of 10.59°, 14.98°, 17.85°, 18.15°, 25.42°, and 26.75° were observable distinctly at unvarying positions in the PMs patterns but with a reduction in their intensities because of the feasible dilution effect of Eudr and PEG 6000. By scrutinizing the previous data, it can be concluded that the crystalline form of GLC was still retained, however, slight diminishment of the intensity of PXRD patterns of GLC in Eudr and PEG 6000 PMs implies that the drug crystalline feature was decreased.^13^ The alterations in intensities of peaks of GLC observed in PXRD patterns of ESSs with both conducted polymers compared to that of PMs could be clarified as a consequence of transforming in crystalline structure (i.e. GLC transformation from a crystalline state to an amorphous phase within the preparation procedure). Furthermore, PEG 6000 PXRD peaks positions in the PM and ESSs were not altered that can be related to a chemical interaction possibleness and a compound establishment between GLC and PEG 6000 (Figure 3b). The results of the current study reveal the presence of GLC in moderately crystalline/nanocrystalline as well as amorphous states in the ESSs. These results have good consistency with DSC and FTIR findings and previously published studies.^13,44^

### Fourier transform infrared spectroscopy

**Figure 4:**
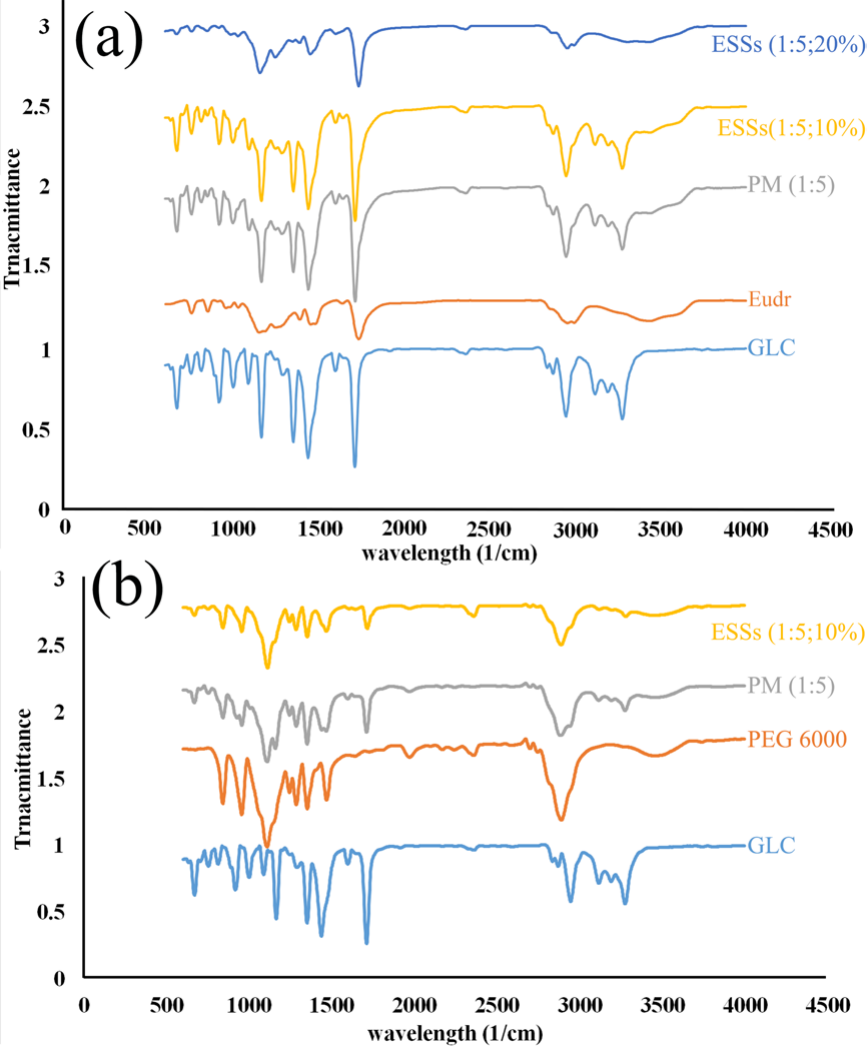
Fourier-transform infrared spectroscopy curves of (a) pure gliclazide (GLC), Eudragit® RS100 (Eudr), physical mixture (PM) and electrosprayed nano-solid dispersions (ESSs) with the drug: polymer ratio of 1:5 at total solution concentrations of 10% and 20% (w/v) and (b) GLC, Polyethylene glycol 6000 (PEG 6000), PM and ESSs 1:5-10% w/v.

FTIR spectroscopy is serviceable whenever attempting to represent compatibility of various components in a sample as well as some interactions as Van der Waals and hydrogen bonding that can be identified by wavelength shifts in FTIR spectra. Figure 4 depicts the FTIR spectrum of the pure drug, Eudr, PEG 6000, PMs and ESSs. The spectroscopic spectrum of GLC exhibited N–H amide bands at 3273, 3192, 1595, and 1164 cm^−1^, C–H aromatic and aliphatic stretches at 3112, 2949, and 2867 cm^−1^, respectively, C=O carbonyl characteristic peak at 1709 cm^−1^, C=C aromatic bands at 1590 and 1473 cm^−1^, S=O sulphonyl stretch at 1348 and 1162 cm^−1^, C–N ring and p-phenyl groups at 1240 and 811 cm^−1^, respectively.^45^ Comparing distinctive peaks of GLC in FTIR spectra of PMs and ESSs in both groups (i.e. samples processed with Eudr and PEG 6000) with that of pure GLC spectrum reveals the presence of these bands with a slight shift and reduced intensity, where this phenomena could be attributed to the dilution effect of the polymers^25,28^ in addition to probability of complexation or hydrogen bonding development between GLC and polymers in solid state.

Figure 3a represents FTIR spectrum of pure Eudr that indicated C-H aliphatic and -C=O stretching peaks at 2991.35 and 1732.64 cm^-1^, respectively. Additionally, PEG 6000 spectrum depicted its significant vibrations including C–H, C–O, and –OH stretching at 2890, 1110, and 3350 cm^−1^, respectively.^13,25^ The aforementioned peaks shift in conjunction with GLC sulphonylurea groups’ might enhance bond strength as a consequence of polymers H_2_ atoms stabilizing influence (due to the interactions between the O_2_ and H_2_ atoms of sulphonyl group and polymers in processed samples)^46^ as a result it can lead to the physical interaction (complexation/hydrogen bonding). These findings are in good conformity with the DSC and PXRD results and other relevant reports.^13,44^

### In vitro dissolution study

**Figure 5:**
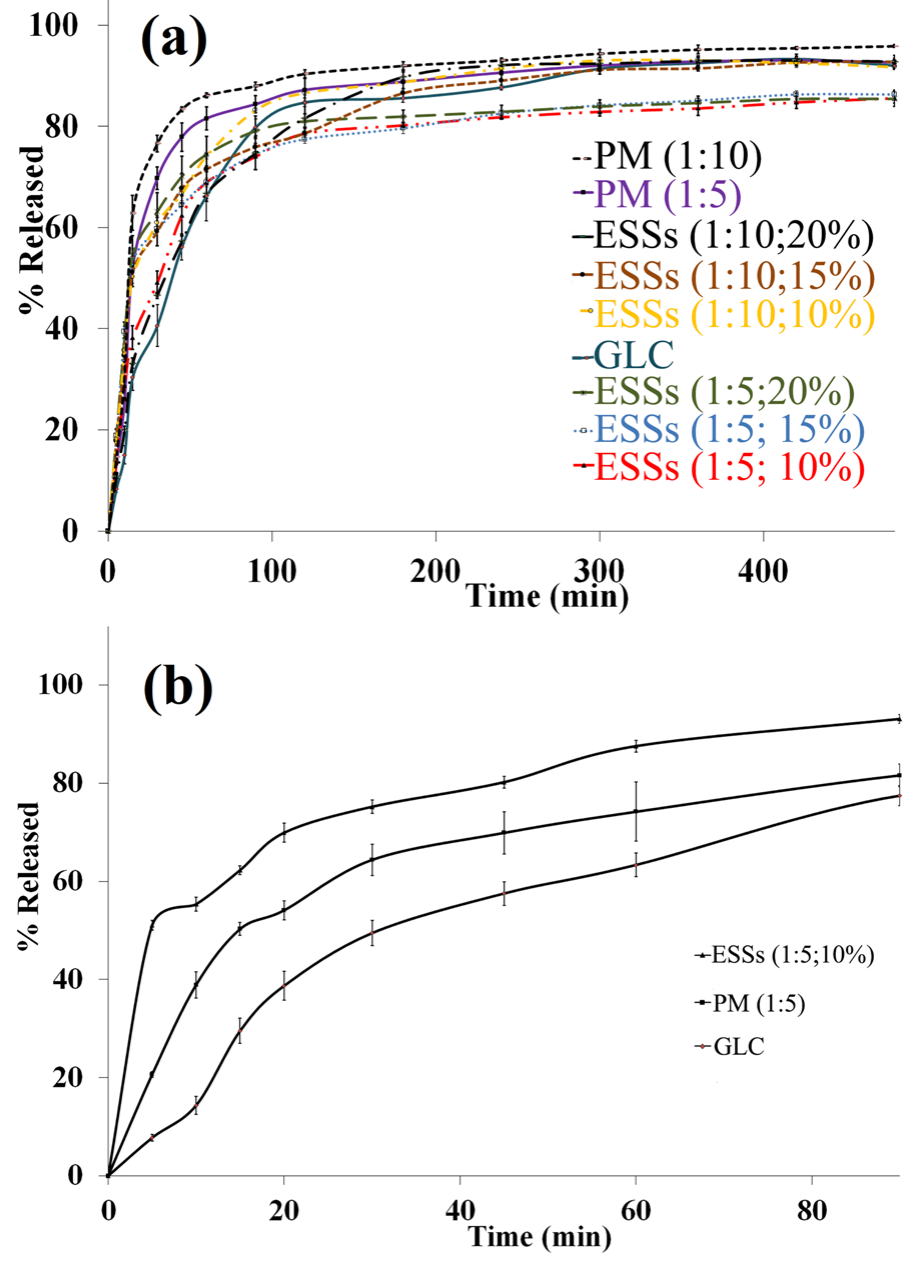
Dissolution profiles of (a) pure gliclazide (GLC), Eudragit® RS100 (Eudr), physical mixture (PM) and electrosprayed nano-solid dispersions (ESSs) with the drug: polymer ratio of 1:5 and 1:10 at total solution concentrations of 10%, 15% and 20% (w/v) and (b) GLC, Polyethylene glycol 6000 (PEG 6000), PM and ESSs 1:5-10% w/v.

Figure 5 indicates the cumulative *in-vitro* release profiles of raw GLC and GLC from PMs and ESSs. The influence of ECS process besides the effects of polymer ratios on the dissolution behaviors of GLC were determined by computing t_50%_ (demanded time for releasing 50% of the drug), Q_30min_ and Q_120min_ (the dissolved drug percent within 30 and 120 min, respectively) magnitudes (Table 3). Moreover, GLC release mechanism form the ESSs was investigated by fitting the release data of these formulations in the six most commonly used models (Table 4). It is obvious that in all the calculated formulations, the Weibull model revealed the highest values of R^2^_adj_ and MSC in comparison with other models proposing the suitability of this model in appropriately fitting the empirical data.

**Table 3:** Computed quantities of the Q_30min_, Q_120min_ and t_50%_, t_60%_ for pure Gliclazide (GLC), physical mixtures (PM) with drug: polymer ratios of 1:5 and 1:10, and electrosprayed nano-solid dispersions (ESSs) with the drug: polymer ratios of 1:5 and 1:10 at total solution concentrations of 10%, 15% and 20% (w/v)

**Formulations prepared using Eudragit** ^®^ **RS100**		
**Formulation**	**t**_50%_	**Q**_120 min_
Gliclazide	30	84.72 ± 0.81
F1 (1:5; 10%)_;_	30	78.59 ± 1.79
F2 (1:5; 15%)	15	77.42 ± 0.83
F3 (1:5; 20%)	15	81.03 ± 1.42
F4 (1:10; 10%)	15	86.61 ± 0.46
F5 (1:10; 15%)	15	78.62 ± 1.23
F6 (1:10; 20%)	30	81.63 ± 2.89
PM (1:5)	15	87.15 ± 2.26
PM (1:10)	8	90.35 ± 0.80
**Formulations prepared using polyethylene glycol 6000**		
	t_50%_	Q_30 min_*
Gliclazide	30	49.47 ± 2.59
F7 (1:5; 10%)	5	75.20 ± 1.37
PM (1:5)	15	64.37 ± 3.19

* Due to the faster release of samples prepared via PEG6000, Q_30min_ was presented. t_50%_ (demanded time for releasing 50% of the drug), Q_30min_ and Q_120min_ (the dissolved drug percent within 30 and 120 min, respectively).

**Table 4:** Release kinetics assessment of electrosprayed nanoformulations with different GLC: polymers ratios at various solution concentrations (%w/v)

**Kinetic model**		**Electrosprayed formulations using Polyethylene Glycol 6000**		**Electrosprayed formulations using Eudragit** ^®^ **RS100**				
		**1:5 10%**	**1:5 10%**	**1:5 15%**	**1:5 20%**	**1:10 10%**	**1:10 15%**	**1:10 20%**
Zero-order	K_0_	0.337	0.291	0.295	0.299	0.323	0.316	0.318
	R^2^_adj_	-2.483	-0.503	-1.051	-1.241	-0.715	-0.669	0.019
	MSC	-2.325	-0.958	-1.422	-1.519	-1.149	-1.128	-0.421
First-order	K_1_	0.070	0.019	0.026	0.031	0.029	0.026	0.018
	R^2^_adj_	0.872	0.816	0.687	0.748	0.916	0.858	0.969
	MSC	0.979	1.139	0.459	0.666	1.867	1.336	3.038
Higuchi	K_H_	6.504	5.419	5.548	5.655	6.039	5.875	5.801
	R^2^_adj_	-0.355	0.583	0.351	0.235	0.502	0.528	0.788
	MSC	-1.381	0.324	-0.272	-0.444	0.087	0.135	1.112
Korsmeyer-Peppas	K_KP_	47.631	21.425	27.941	30.537	26.411	25.344	16.327
	n	0.126	0.244	0.198	0.185	0.225	0.227	0.308
	R^2^_adj_	0.924	0.906	0.922	0.884	0.916	0.931	0.922
	MSC	1.431	1.747	1.782	1.375	1.807	2.000	2.046
Hixson-Crowell	K_HC_	0.004	0.003	0.004	0.004	0.004	0.004	0.004
	R^2^_adj_	-0.281	0.609	0.327	0.266	0.575	0.558	0.867
	MSC	-1.325	0.389	-0.308	-0.403	0.247	0.201	1.575
Weibull	α	3.203	4.802	2.847	2.610	4.343	3.948	11.444
	β	0.447	0.394	0.295	0.291	0.437	0.394	0.614
	T_i_	0.000	4.595	4.832	4.880	4.315	4.507	3.743
	R^2^_adj_	0.975	0.981	0.995	0.983	0.995	0.995	0.995

Note: K_0_, K_1_, KH, KKP, n, KHC, α, β, T_i_: The parameters of the studied models, R^2^_adj_: The adjusted coefficient of determination, MSC: The model selection criterion (MSC).

Considering Figure 5a and the data related to SDs prepared using Eudr as the carrier (Table 3), it is clear that in comparison with pure GLC and PMs the ESSs depicted slightly slower dissolution rates at the identical pH. The drug and polymer compositions are a key parameter that could significantly impress the dissolution behaviors of a drug from electrosprayed formulations.

It should be minded that between the drug and polymer molecular chains, complex phenomena may develop, including the drug attachment to the polymeric carrier surface caused by the electrostatic forces and its entrapment inside the polymeric chains.^25,32^ For case in a point, the release of the drug takes place from a swellable polymeric composition in the ESSs, demanding the attached drug desorption from the surface of the hydrophilic polymer, its diffusion through the polymeric carrier and the polymer swelling. These are the reasons could explain the observed slow release of GLC from ESSs in the current study.

The release profiles in Figure 5b shows that in spite of the SDs prepared using Eudr as polymeric matrix, the SDs formulated applying PEG 6000 had considerably faster drug release rate than the PM and pure GLC. Suggested feasible mechanisms for the increment of the drug release kinetics from PEG 6000 SDs are (I) the polymer solubilization influence, (II) the dissolution of GLC in the hydrophilic polymer, (III) establishment of a uniform drug layer and (IV) the drug release from an immense surface area.^13^ Besides, it has been reported that surface characteristics modification and consequently decreasing the contact angle which augments the drug wettability should induce boost dissolution rates. Wettability improvement of the drug could be achieved by a PEG 6000 film formation around the drug that reduces its surfaces hydrophobicity.^13,47^ Further dissolution rates augmentation mechanisms of SDs may be related to size reduction or aggregation absence of drug crystallites, dispersibility enhancement of the drug, and phase transformation of the drug from the crystalline phase to the amorphous state.^19,34,35^

Advantages of the amorphous electrosprayed SDs could be understood well if one considers the enthalpic energy. Three principal amounts (i.e. crystalline lattice force, cavitation force and solvation force) ruling a drug solubility in a solution. The crystalline lattice force designates for the required energy to interrupt the crystalline structure and eliminate detached molecules. The cavitation force indicates the needed energy for producing a cavity by disturbing water to accommodate the solute molecule in solution. The solvation force defines the energy release after developing beneficial interactions between the solute and solvent. Generally, the solvation and cavitation forces are lower than crystal lattice force and therefore it should overcome this force to propelling the solubility.^48^ The aim of developing an amorphous SDs is to reduce this force partition by destructing the drug crystalline structure in the delivery stage. As a result of the aforementioned mechanisms it can be concluded that the dissolution rate of GLC from PEG 6000 ESSs was increased because of the wettability improvement of GLC and its nanocrystalline as well as amorphous states formation.

The Weibull model was derived from empirical data, so this model conventionally have been benefited to assess the release kinetics of various formulations.^49^ Although the used parameters in this model have not physical nature, but its shape parameter (β) magnitude can reveal the drug transport mechanism within the polymeric network and various values of β stands for different release behaviors. Where, Fickian diffusion is the dominant release mechanism when β <0.75, while a contribution of Fickian diffusion and swelling is predicted for values of 0.75 < β < 1. The first-order kinetics rule the drug release in β = 1 and values of β > 1 contributed to a complex release mechanism.^50-52^ The calculated values of β in the current study (Table 4) were less than 0.75 in all the ESSs, revealing that the Fickian diffusion was dominant release mechanism of GLC from the polymeric networks.

## Conclusion

Electrospraying as an emerging alternative method for SD preparation was effectively conducted to formulate GLC (as a poorly water soluble drug) and enhance its dissolution rate using two kinds of hydrophilic polymers (i.e. Eudragit^®^ RS100 and PEG 6000) at various drug: polymer ratios (i.e. 1:5 and 1:10) with different total solution concentrations of 10%, 15% and 20% *w/v*. The microstructure analysis demonstrated that the drug: polymer ratios together with the total solution concentrations alteration remarkably impressed physical characteristics of the electrosprayed SDs, in which the beads/particles size augmented, increasing the solution concentration and the highest concentration led to the fibers formation. DSC Thermograms of the electrosprayed SDs as well as PXRD and FTIR results suggested that GLC is entirely solubilized in the applied polymers, or its crystalline structure transformed to an amorphous state. In accordance with the in vitro drug release analyses, although the electrosprayed SDs formulated using Eudr as the carrier were not depicted meaningfully faster drug release rate than the PMs and pure drug, but the dissolution rate of GLC from PEG 6000 ESSs was significantly augmented probably because of the wettability improvement of GLC and its nanocrystalline as well as amorphous states formation within the ECS procedure. Fickian diffusion was the dominant mechanism of GLC transportation through the polymer matrices based on kinetic assessments. Our study displayed that the ECS as a productive, novel, and straight forward approach could be practically utilized to prepare formulations of GLC with better physicochemical attributes.

## Ethical Issues

Not applicable.

## Conflict of Interest

The publication has been approved by all co‐authors and the responsible authorities at the institute(s) where the work has been carried out.

## Authors contribution

KA conceived the original idea, supervised the project, designed the experiments, and aided in interpretation of data. SG did the experiments and collected the data. KOB analysed the data, presented data, and drafted and revised the manuscript. NB contributed to the experiments and collected the data. SE contributed to data analysis, data presentation, and writing and reviewing of the manuscript. MBJ contributed to the study consultation, conceptualization of the manuscript, and to the overall writing and editing of the manuscript. All authors discussed the contents and contributed to the final manuscript.

## Acknowledgments

This study was financially supported by the Vice Chancellor for Research of Tabriz University of Medical Sciences grant (No. 3977), Tabriz, Iran.
